# Complementary Sequential Circulating Tumor Cell (CTC) and Cell-Free Tumor DNA (ctDNA) Profiling Reveals Metastatic Heterogeneity and Genomic Changes in Lung Cancer and Breast Cancer

**DOI:** 10.3389/fonc.2021.698551

**Published:** 2021-07-16

**Authors:** Say Li Kong, Xingliang Liu, Swee Jin Tan, Joyce A. Tai, Ler Yee Phua, Huay Mei Poh, Trifanny Yeo, Yong Wei Chua, Yu Xuan Haw, Wen Huan Ling, Raymond Chee Hui Ng, Tira J. Tan, Kiley Wei Jen Loh, Daniel Shao-Weng Tan, Quan Sing Ng, Mei Kim Ang, Chee Keong Toh, Yi Fang Lee, Chwee Teck Lim, Tony Kiat Hon Lim, Axel M. Hillmer, Yoon Sim Yap, Wan-Teck Lim

**Affiliations:** ^1^ Cancer Therapeutics and Stratified Oncology, Genome Institute of Singapore, Singapore, Singapore; ^2^ Clearbridge mFluidics Pte Ltd, Singapore, Singapore; ^3^ Department of Biomedical Engineering, National University of Singapore, Singapore, Singapore; ^4^ Department of Anatomical Pathology, Singapore General Hospital, Singapore, Singapore; ^5^ Division of Medical Oncology, National Cancer Center, Singapore, Singapore; ^6^ Duke-NUS Medical School, Singapore, Singapore; ^7^ Biolidics Ltd, Singapore, Singapore; ^8^ Institute of Pathology, Faculty of Medicine and University Hospital Cologne, University of Cologne, Cologne, Germany; ^9^ Center for Molecular Medicine Cologne, University of Cologne, Cologne, Germany; ^10^ IMCB-NCCS-MP Singapore OncoGenome Laboratory, Institute of Molecular and Cell Biology, Singapore, Singapore

**Keywords:** circulating tumor cells, cell-free tumor DNA, amplicon-sequencing, metastatic signatures, genomic heterogeneity, evolving alterations, lung cancer, breast cancer

## Abstract

**Introduction:**

Circulating tumor cells (CTCs) and cell-free tumor DNA (ctDNA) are tumor components present in circulation. Due to the limited access to both CTC enrichment platforms and ctDNA sequencing in most laboratories, they are rarely analyzed together.

**Methods:**

Concurrent isolation of ctDNA and single CTCs were isolated from lung cancer and breast cancer patients using the combination of size-based and CD45-negative selection method *via* DropCell platform. We performed targeted amplicon sequencing to evaluate the genomic heterogeneity of CTCs and ctDNA in lung cancer and breast cancer patients.

**Results:**

Higher degrees of genomic heterogeneity were observed in CTCs as compared to ctDNA. Several shared alterations present in CTCs and ctDNA were undetected in the primary tumor, highlighting the intra-tumoral heterogeneity of tumor components that were shed into systemic circulation. Accordingly, CTCs and ctDNA displayed higher degree of concordance with the metastatic tumor than the primary tumor. The alterations detected in circulation correlated with worse survival outcome for both lung and breast cancer patients emphasizing the impact of the metastatic phenotype. Notably, evolving genetic signatures were detected in the CTCs and ctDNA samples during the course of treatment and disease progression.

**Conclusions:**

A standardized sample processing and data analysis workflow for concurrent analysis of CTCs and ctDNA successfully dissected the heterogeneity of metastatic tumor in circulation as well as the progressive genomic changes that may potentially guide the selection of appropriate therapy against evolving tumor clonality.

## Introduction

Tissue biopsies and radiological imaging are routinely utilized by clinicians to monitor treatment efficacy and disease progression in patients. However, tissue biopsies are invasive and often associated with risks and pain or discomfort, while frequent imaging for example with computed tomography may be costly and involves cumulative exposure to ionizing radiation. Furthermore, tissue biopsy may be limited by sampling error as single site biopsy may not reflect the profile of the whole tumor. In contrast, minimally invasive liquid biopsy allows repetitive non-invasive sample collection and offers a broader tumor genomic profile based on shed DNA or cells from the tumor, with potential for better real-time monitoring of treatment efficacy and disease progression.

The metastatic spread of cancer is largely due to the shedding of circulating tumor cells (CTCs) from tumors into the blood stream and invasion of distant organs. Characterization of CTCs has provided mutation profiles of emerging tumor subclones that contribute to metastatic spread and resistance to therapy (1, 2). The number of CTCs detected in the blood of cancer patients also correlates with inferior treatment response and survival outcomes (3), suggesting that the assessment of CTCs have prognostic and predictive importance in monitoring treatment efficacy (4). However, beyond the quantification of CTCs, molecular analysis of CTCs is essential for improving our understanding of the tumor biology which may in turn have therapeutic implications.

Recently, much attention and effort have been focused on utilizing cell free tumor DNA (ctDNA) as liquid biopsy because of its greater accessibility and easier utility. However, caveats remain as ctDNA consists of fragmented DNA from apoptotic and necrotic tumor cells shed into the bloodstream (5), hence it provides limited insight to tumor biology of individual cells. Moreover, genetic signatures obtained from ctDNA are derived from the major clone in the tumor. The signatures of subclonal tumor that do not respond to the treatment and drive disease progression could be missed by ctDNA (6). Hence, additional complementary information from CTCs may be useful.

Due to the limited access to both CTC enrichment platforms and ctDNA sequencing in most laboratories, parallel analysis of CTCs and ctDNA is rare. Furthermore, the technology for isolation of rare CTCs at single cell resolution is restricted to costly and complex platforms. In this study, we utilized the single cell isolation platform, DropCell for CTC isolation at single cell resolution. We implemented a standardized sample processing workflow that allowed for concurrent isolation of CTC and ctDNA followed by targeted amplicon sequencing to evaluate the genomic heterogeneity of ctDNA and CTCs in lung and breast cancer patients.

## Materials and Methods

### Patients

A total of 16 lung adenocarcinoma and 21 breast ductal carcinoma patients diagnosed at the National Cancer Centre Singapore were recruited. A total of 48 paired tumor tissues were obtained from the surgical resections/biopsies at disease diagnosis and progression. The detailed description of the patient’s sample can be obtained in **Supplementary Table 1**.

### Separation of Plasma and Buffy Coat for Isolation of ctDNA and CTCs

A total of 10ml EDTA blood was collected from the patients. We allocated 2.5ml blood for collection of buffy coat as germline DNA control sample, while 7.5ml blood was subjected to CTC enrichment using ClearCell^®^ FX1 (Biolidics) platform following the manufacturer’s recommendation. First, the plasma was isolated by low-speed centrifugation at 500g for 10 minutes. The supernatant was transferred to another 1.5 ml Eppendorf tube followed by high speed centrifugation at 16,000g for 10 minutes at 4°C. The supernatant was snap frozen in dry ice and stored at -80°C. Second, the red blood cells were lysed using RBC lysis buffer (G-Biosciences) in a 1 blood: 3 buffer ratio for 10 minutes. The buffy coat was collected by centrifugation at 500g for 10 minutes. The supernatant was discarded and the buffy coat pellet was resuspended in 4mL of ClearCell FX Resuspension Buffer (Biolidics), followed by CTC enrichment on ClearCell^®^ FX1 platform using protocol 1.

### Single Cell Isolation With DropCell

In order to isolate CTCs at single cell resolution, we applied CD45-antibody negative selection using a single cell capture method as described previously (7, 8). In brief, larger cells retrieved from the buffy coat were processed through the single cell selection microfluidics. Cells were pre-incubated with CD45-FITC antibodies (Abcam Inc., USA) and positively fluorescent cells were excluded. Cells that fit the criteria of large nucleus to cytoplasmic (N/C) ratio, intact cell membrane and CD45-negative fluorescent stains were ejected from the microfluidics platform one at a time for single cell processing.

### DNA Extraction of Formalin-Fixed Paraffin-Embedded (FFPE) Tissues

DNA was extracted from the FFPE samples using the GeneRead DNA FFPE Kit (Qiagen). Following the manufacturer’s instructions, each section of the FFPE sample underwent deparaffinization and digestion, before allowing the DNA to bind to the QIAamp MinElute column. While bound, the DNA was washed to remove any contaminants. The DNA was then eluted with 25μl of Buffer ATE.

### DNA Extraction of Frozen Tissues

DNA was extracted from frozen tissue using the AllPrep DNA/RNA/miRNA Universal kit (Qiagen). Following the manufacturer’s instructions, the frozen tissue was first disrupted using the mortar and pestle method, before being homogenized using the QIAshredder (Qiagen). The homogenized lysate was spun in an AllPrep DNA Mini spin column, where DNA was bound to the column and the RNA was found in the flow-through. Total RNA was first purified and bound on the RNeasy mini spin column. While bound, the RNA was washed to remove any contaminants. RNA was eluted in 30μl of RNase-free water. Next, genomic DNA that was bound on the AllPrep DNA Mini spin column was washed to remove any contaminants. 50μl of Buffer EB was used to elute the DNA.

### DNA Extraction of Circulating Cell-Free Nucleic Acid

DNA was extracted from plasma using the QIAamp Circulating Nucleic Acid kit (Qiagen). Following the manufacturer’s instructions, the sample was first lysed to release DNA bound to proteins, before allowing it to bind to the QIAamp Mini column using the VacConnector on the QIAvac 24 Plus. While bound, the DNA was washed to remove any contaminants. The DNA was eluted with 30μl of Buffer AVE.

### Whole Genome Amplification for Lung CTC Samples

Whole genome amplification was performed on the isolated single lung CTC samples using REPLI-g single cell kit (Qiagen). The denaturation buffer was added to the DNA followed by a 3 min incubation at room temperature. The denaturation was terminated by addition of neutralization buffer. The DNA amplification was performed in a reaction mix consisting of reaction buffer and DNA polymerase for 1.5 hours at 30°C. The reaction was terminated by inactivation of the DNA polymerase at 65°C for 3 min. The amplified DNA was cleaned using ethanol precipitation.

### Quality Control of Amplified Lung CTC With Quantitative PCR

In order to evaluate the quality of the amplified DNA in particularly the possible allelic dropout rate during WGA, we designed a total of 10 primers sets targeting genomics regions of different chromosomes (**Supplementary Table 2**). The qPCR reactions were carried out using SYBR Green Master Mix (Roche) with 10ng of input DNA and 0.6nM of primers (IDT) on the LightCycler^®^ 480 platform (Roche) with the following thermocycling conditions: 95°C for 5 mins; 45 cycles of 95°C for 10s and 60°C for 1 min; 95°C for 10s and 65°C for 1 min. Samples with Ct values <35 in at least 5 of the 10 target regions (50% positive rate) were selected for downstream DNA sequencing experiment.

### Whole Genome Amplification for Breast CTC Samples

Whole genome amplification was performed on the isolated single breast CTC samples using Ampli-1 Single Cell WGA kit (Silicon Biosystems). The cell lysis was performed with the addition of Lysis Reaction Mix to each sample. DNA digestion was carried out with the Digestion Reaction Mix followed by ligation with Ligation Reaction Mix. PCR was carried out following the recommended thermo-cycling condition.

### Quality Control of Amplified Breast CTC With Quantitative PCR

In order to evaluate the quality of the amplified DNA, we performed PCR experiment on the amplified DNA with Ampli1 QC Kit (Silicon Biosystems) following the manufacturer’s recommendation. Samples that gave positive amplification for at least 2 out of the 4 amplicons (50% positive rate) were selected for downstream amplicon-sequencing work.

### Allele Specific PCR (ASPCR)

The *EGFR* T790M and *EGFR* exon19 deletion mutation assay were purchased from Life Technologies. ASPCR was conducted on ABI7900 qPCR machine (Life Technologies) using 1x TaqMan Genotyping Master Mix, 1x TaqMan Probe and 20ng of DNA with the following thermal-cycling conditions: 95°C for 10 mins; 5 cycles of 92°C for 15s and 58°C for 1 min; 40 cycles of 92°C for 15s, 60°C for 1 min.

### Sanger Sequencing

PCR for detection of *EGFR* Exon19 deletion mutation was carried out using 0.4µM primers: Forward: 5’-ATGTGGCACCATCTCACAAT-3’; Reverse: 5’-CAGCTGCCAGACATGAGAAA-3’; 20ng of DNA, 1x AccuTaq LA Buffer (Sigma), 500µM dNTP, 0.05U/µl JumpStart RED AccuTaq LA DNA Polymerase under the following thermo-cycling condition: 94°C for 3 mins; 15 cycles of 94°C for 20s, 58°C for 30s, 68°C for 1 min; 20 cycles of 94°C for 20s, 55°C for 30s, 68°C for 1 min; 68°C for 5 mins. The PCR amplicon was purified with PCR purification kit (Qiagen) following the recommended protocol. The purified PCR product was submitted to Sanger sequencing service provider, Axil Scientific.

### GeneRead Targeted DNAseq

We customized 2 different gene panels using Qiagen’s GeneRead DNAseq Custom Builder tool. The list of genes and its coverage regions are listed in **Supplementary Tables 3, 4**. Multiplex PCR was performed using GeneRead HotStar Taq DNA polymerase and four primer pools with a total of 80 ng of tumor or CTC DNA and 20ng of cell-free plasma DNA. The amplicons were pooled together and cleaned using AMPure beads (Beckman Coulter). The PCR-enriched DNA was subjected to next-generation sequencing library construction using QIAseq 1-Step Amplicon Library Kit (Qiagen). Each library was barcoded with a unique index and quantified using KAPA Library Quantification kit (Kapa Biosystems). Equal amounts of individual libraries were pooled together for a 150bp paired-end sequencing run on the Nextseq (Illumina) platform.

### Reads Alignment and Base Quality Refinement

The sequenced reads were mapped to the human reference genome hg19 using BWA pipeline (0.7.12) followed by pre-process based on Genome Analysis Toolkit (GATK 3.5) (9) best practices and BAQ (Base Alignment Quality) calculation using SAMtools (1.3) (10). The aligned reads were sorted based on coordinates.

### Variant Calling and Copy Number Variation Analysis

We used LoFreq 2.1.1 (11) pipeline for detection of single nucleotide variant (SNV) and insertions/deletions (INDEL) variants with default parameters. We used Quandico 1.13 for copy number variation (CNV) detection with the following modification to the default setting: primer length was set to 21 (average primer length of our GeneRead panel); reads with mapping quality score less than 30 were excluded from the analysis and we grouped the reads into regions as qcluster.

### The Analytical Pipeline for Detection of Mutation and Copy Number Variation

**Figure 1:**
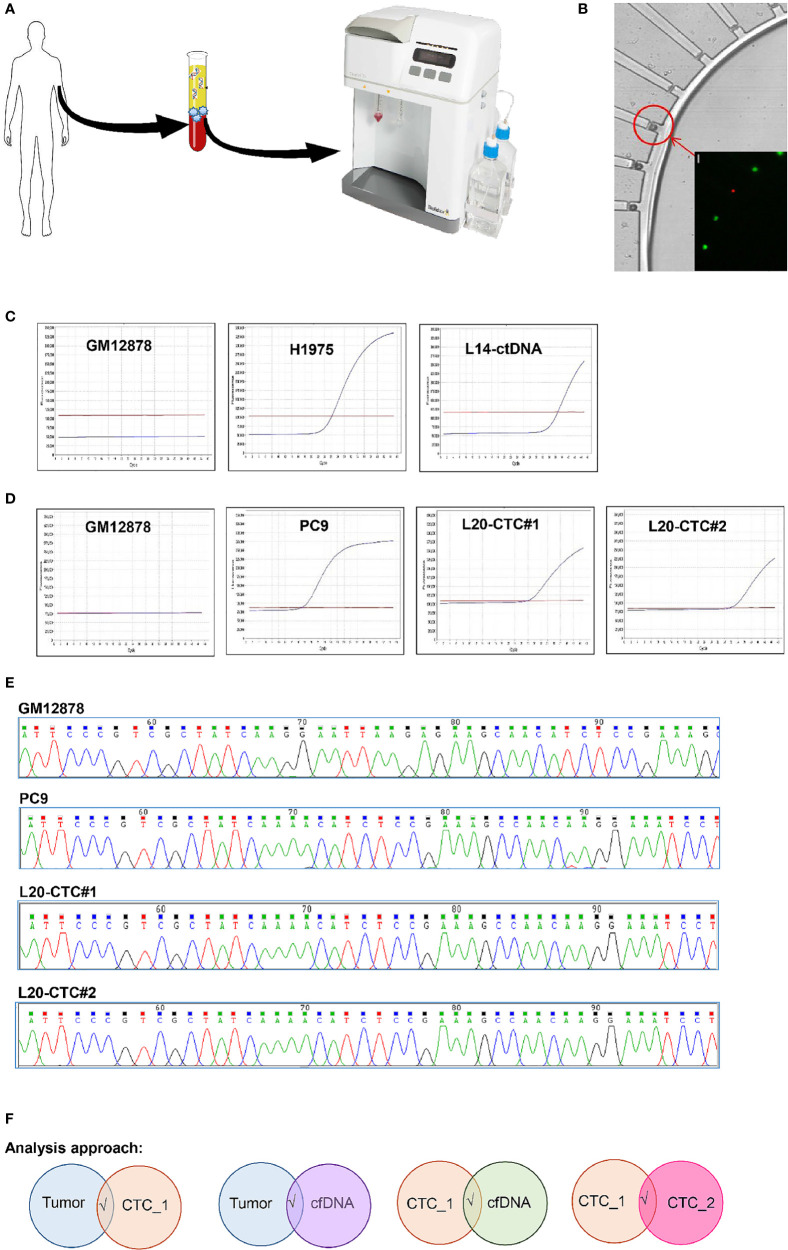
The workflow for sample’s isolation, CTC mutation validation and analysis pipeline used in this study. **(A)** The isolation of CTCs and ctDNA from the same tube of blood. CTC was enriched through the ClearCell FX system. **(B)** CTC was isolated on single cell resolution using the DropCell platform. An image of the cells captured in the cell chamber. **(C)** The detection of *EGFR* T790M mutation with ASPCR in the negative control (GM12878 cell); positive control (H1975 cell) and ctDNA from a lung cancer patient. **(D)** The presence of *EGFR* Exon19 deletion mutation with ASPCR in the negative control (GM12878 cell); positive control (PC9 cell) and two CTCs isolated with the DropCell platform from a lung cancer patient. **(E)** The electropherogam of Sanger sequencing run for validation of *EGFR* Exon19 deletion mutation in negative control (GM12878 cell); positive control (PC9 cell) and two CTCs from a lung cancer patient with known *EGFR* Exon19 deletion mutation. **(F)** The analysis pipeline used in this study.

A reliable analytical pipeline to minimize false positive detection of mutation in our data was developed to address random errors associated with whole genome amplification and sequencing (12), as well as sequencing error/variation due to low abundance of ctDNA samples. Our previous work has conducted a systematic evaluation of the amplification error generated during whole genome amplification using normal DNA as test sample (1). We demonstrated that applying a 10% cut-off on the variant allelic frequency (VAF) of amplified DNA, it could avoid false positive detection of mutation owing to amplification errors. Hence, we applied the same 10% VAF threshold for single CTC that has undergone DNA amplification, and 1% VAF for ctDNA and tumor samples. Further, in order to eliminate false positive detection without missing out genomics alteration due to tumor heterogeneity, we only considered mutations that met the following criteria: shared between tumor and CTC or ctDNA samples; shared between CTC and ctDNA samples; shared between at least two individual CTCs from the same patient (**Figure 1F**).

### Survival Analysis

The survival analysis were carried out for top five genes that were altered in lung cancer samples (*CSMD2, DMBT1, EGFR, RYR2* and *NOTCH1)* and breast cancer samples (*MTOR, KMT2C, EGFR, ERBB3* and *USH2A)* using the cBioPortal (13, 14) survival analysis tool from a total of 511 lung cancer and 996 breast cancer patients cohort data (15–21).

## Results

### Establishing a Systematic Workflow for Isolation of ctDNA and Single CTCs

We obtained single time point clinical specimens from 16 lung cancer and 5 breast cancer patients from a single comprehensive cancer centre. In addition, serially collected tumors and blood samples were collected from another 16 breast cancer patients. The clinicopathological information of these patients are described in **Tables 1**, **2**. The baseline specimens were collected at baseline prior to initiation of specific line of systemic anti-cancer therapy. Additional blood samples were collected after two cycles while on therapy where feasible, as well as at first response assessment and eventually at disease progression or completion of therapy.

**Table 1:** The clinicopathological features of lung cancer patients recruited in this study.

Clinicopathlogic feature	Details	n	%
Age at diagnosis	<60	8	50
	≥60	8	50
	Median	60.5	
	Range	35-84	
Disease stage at the point of blood collection	Stage I	0	0
	Stage II	1	6.25
	Stage III	4	25.00
	Stage IV	11	68.75
Smoking History	Yes	3	18.75
	No	10	62.5
	Unknown	3	18.75

**Table 2:** The clinicopathological features of breast cancer patients recruited in this study.

Clinicopathlogic feature	Details	n	%
Age at diagnosis	<50	9	42.9
	≥50	12	57.1
	Median	54	
	Range	27-65	
Disease stage at the point of blood collection	Stage I	0	0
	Stage II	2	9.5
	Stage III	5	23.8
	Stage IV	14	66.7
ER/PR/HER2	ER+ and/or PR+, HER2-	9	42.9
	HER2+	9	42.9
	ER-/PR-/HER2-	3	14.3
CA15.3	≤25.1 U/mL	9	42.9
	>25.1 U/mL	10	47.6
	Not tested	2	9.5
	Median	37.3	
	Range	12.4-1341	

CTCs display dynamic phenotypic changes from epithelial to mesenchymal and vice versa during the different stages of disease progression (22). Here, we utilized a combination of size-based and CD45-negative selection method to enrich for CTCs In order to ensure sample comparison validity, CTCs and ctDNA were isolated from the same blood tube. The buffy coat component was used to isolate single CTCs using ClearCell FX and DropCell platforms while the plasma portion was used in ctDNA detection (**Figures 1A, B**). A total of 116 CTCs and 41 plasma were collected from lung cancer patients while 159 CTCs and 53 plasma were obtained from breast cancer patients (**Supplementary Table 5**). Upon implementing the analytical pipeline to eliminate the whole genome amplification and sequencing errors, we kept a total of 16 tumors, 39 CTCs and 14 ctDNA from 16 lung cancer patients; a total of 32 tumors, 108 CTCs and 35 ctDNA from 21 breast cancer patients in the final data set.

We detected the presence of *EGFR* T790M mutation in the isolated ctDNA (**Figure 1C**) as well as the *EGFR* exon 19 deletion using allelic-specific PCR (ASPCR) and Sanger sequencing on single cells isolated from a lung cancer patient with known *EGFR* exon 19 deletion mutation (**Figures 1D, E**) using this workflow, proving the capability of the technology for this purpose that is in concordant with previously reported studies from other cohorts of lung and breast cancer patients (7, 23).

### The Molecular Profiling of the Tumours, CTCs and ctDNA

In order to assess the mutation profiles of CTCs, ctDNAs and tumors, we custom-designed targeted gene panels for amplicon-sequencing. The lung cancer GeneRead panel consisted of the 45 most frequently mutated or druggable genes in lung cancer as previously reported (24, 25) with a targeted region of ~207kb. The breast cancer GeneRead panel consisted of the 58 most frequently mutated or druggable genes in breast cancer as reported by TCGA (26) with a targeted region of ~233kb.

**Figure 2:**
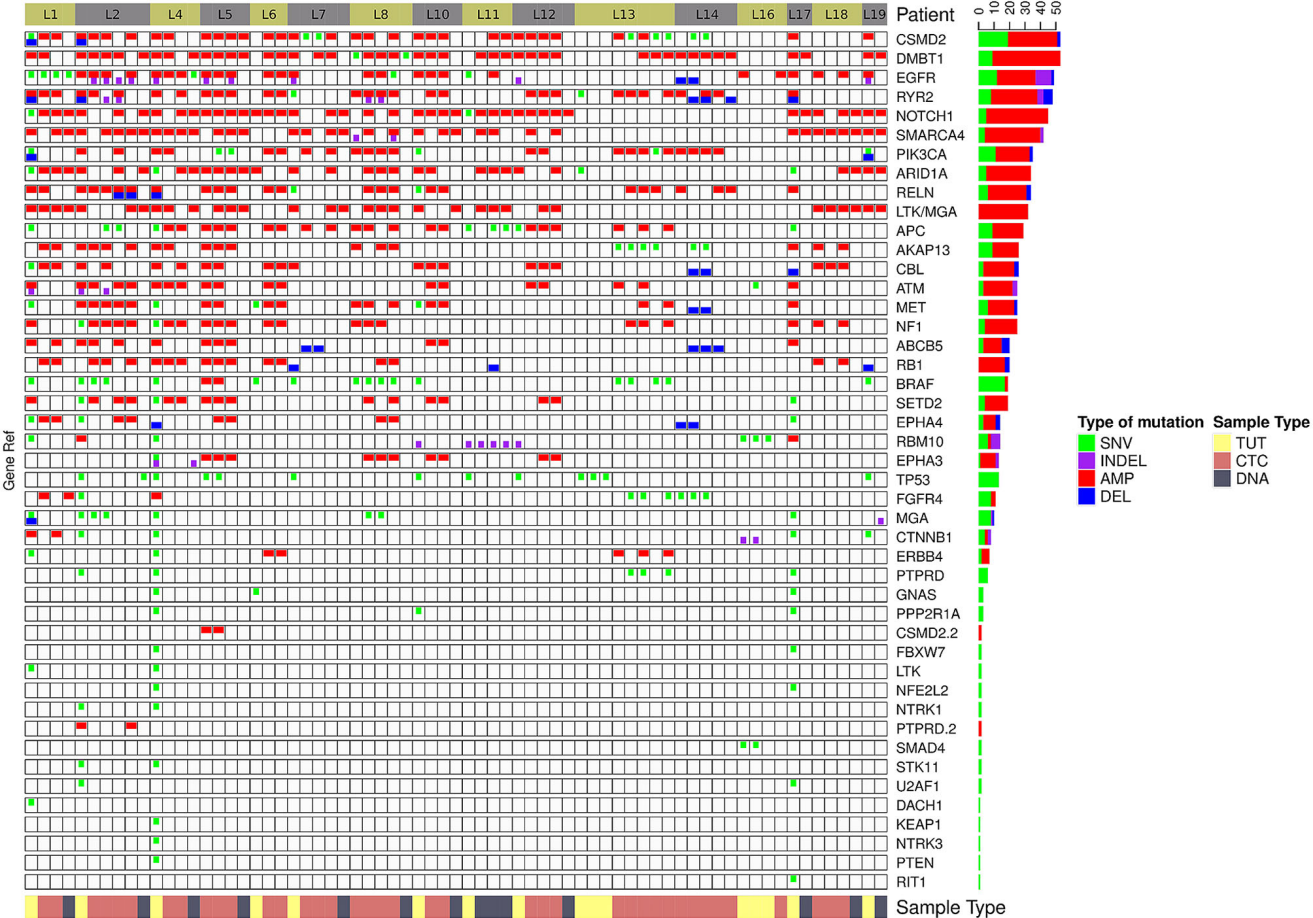
The genomics alteration detected in the lung cancer samples. The tabulation of exonic SNVs, in-frame or frame-shift INDELs and CNVs of frequently mutated genes. The right panel displays the cumulative numbers of alterations for individual genes. Patient’s sample IDs are shown at the top panel. The bottom panel shows the type of samples analyzed. Red rectangles represent amplifications. Blue rectangles represent deletions. Green rectangles represent missense, Stopgain or Stoploss somatic SNVs. Purple rectangles represent somatic INDELs. TUT, CTC and DNA refer to tumors, individual CTC and ctDNA respectively.

**Figure 3:**
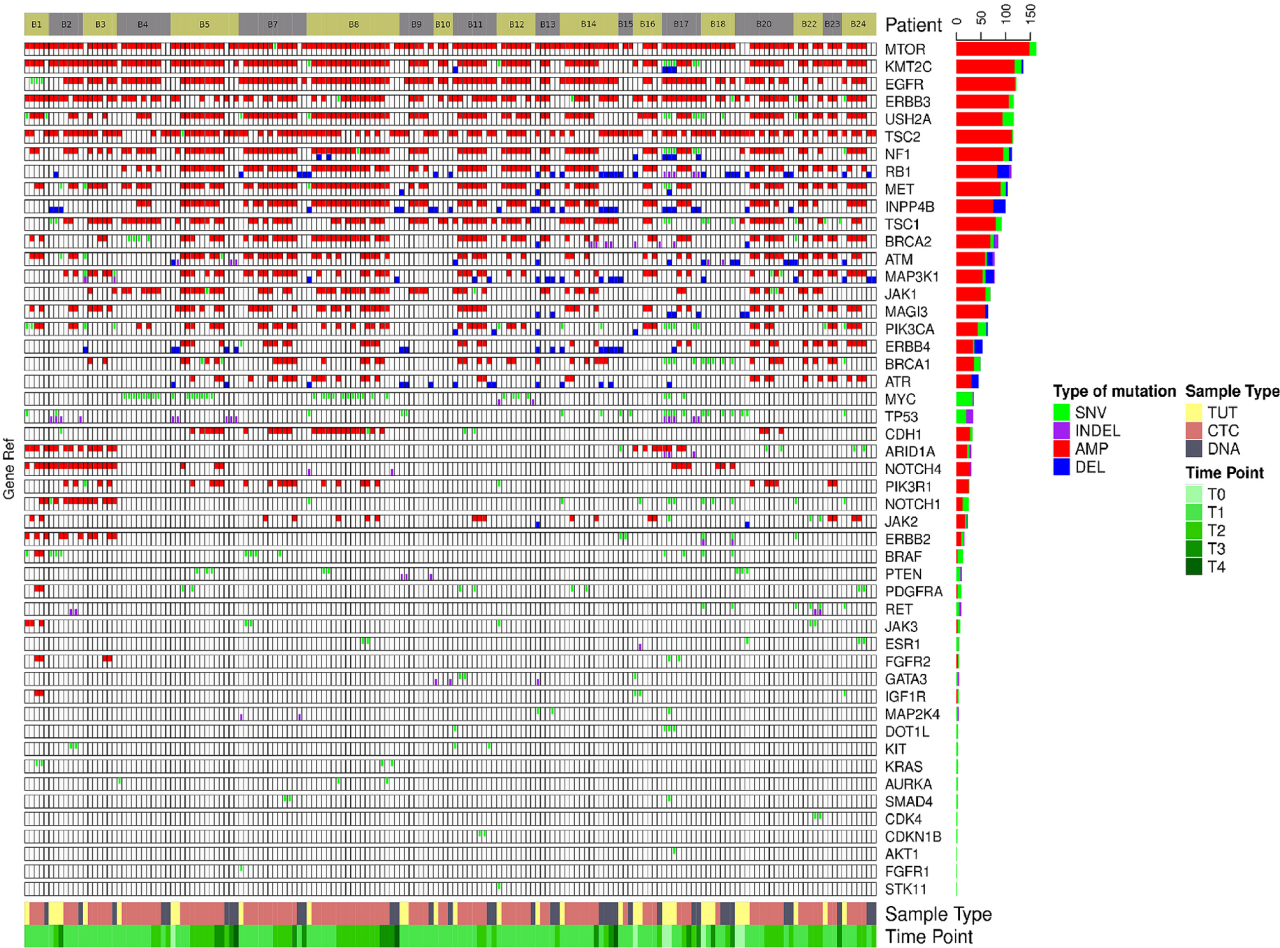
The genomics alteration detected in the breast cancer samples. The rectangle panels and colors are as described in **Figure 2**. In addition, the time point of the serially collected samples for selected patients are represented by the different tones of green color at the bottom panel from 1^st^ (T1) up to 4^th^ time point (T4) during the course of this study. T0 indicates the time point that the tumor was collected before this study.

We found that single nucleotide variation (SNV) or insertion/deletion (INDEL) mutations were frequently detected in *EGFR, CSMD2, BRAF, TP53* and *RYR2* genes in lung cancer samples (**Figure 2** and **Supplementary Table 6**), while frequent copy number alterations were found in *RYR2, RELN, DMBT1, CSMD2 and NOTCH1* (**Supplementary Table 7**). In contrast, we noted that the SNV or INDEL mutations were commonly detected in *MYC, TP53, USH2A, NOTCH1 and PIK3CA* genes in breast cancer samples (**Supplementary Table 8**), while *MTOR, KMT2C, EGFR, USH2A* and *NF1* amplification were frequently found in breast cancer samples (**Figure 3** and **Supplementary Table 9**).

### The Heterogeneity of Genomic Alterations in Tumour, CTCs and ctDNA

**Figure 4:**
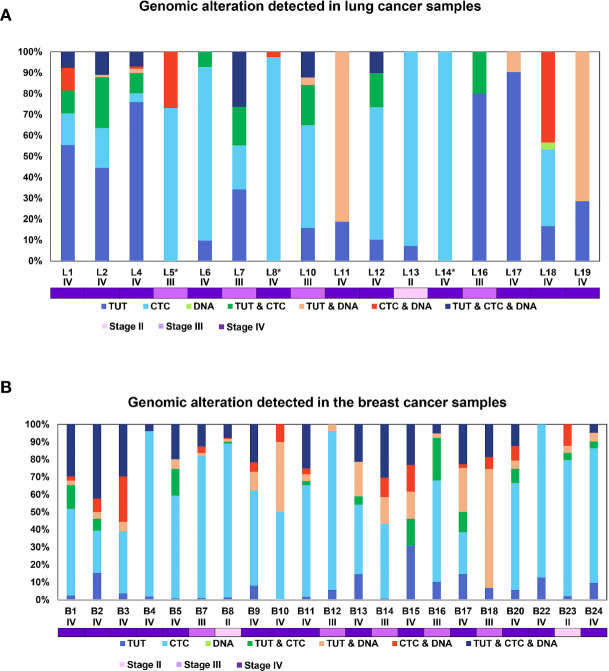
The configuration of the genomic alterations detected in **(A)** lung and **(B)** breast cancer samples. The mutations are grouped according to their occurrence, either private or commonly found in tissue, CTCs or ctDNA. The disease stage of each patient is represented by the different tones of purple colors.

Though intratumoral heterogeneity (ITH) has been well studied in lung and breast cancers (25, 27–29), the comparative degree of heterogeneity between the CTCs, ctDNA and tumor remains poorly described. The distribution of the genomic alterations present in all the corresponding tumors, CTCs and ctDNAs collected for each patient are shown in **Figure 4**. We observed a high degree of heterogeneity in the mutation profiles of CTCs that were undetected in the matched tumor. We found that 78% of the lung CTCs and 91% of the breast CTCs had at least one shared mutation with the matched tumors (**Tables 3**, **4**). In contrast, we observed that all (100%) of the lung and breast ctDNA have at least one shared genomic alteration with the matched tumor (either primary tumor and/or metastatic lesion). Moreover, we noted that some mutations shared between CTCs and ctDNA were absent in the tumor. These findings highlight the heterogeneity of tumor subclones shed into circulation.

**Table 3:** The tabulation of the mutations shared between tumor, CTCs and ctDNA for lung cancer samples.

Patient	Disease stage at the point of blood collection	Shared mutations between Tumor and CTC	Shared mutations between Tumor and ctDNA	Shared mutations between CTC1 and CTC2	Shared mutations between CTC and ctDNA	Availability of tumor
L1	IV	9	2	5	4	Yes
L2	IV	21	6	12	0	Yes
L4	IV	12	6	5	1	Yes
L5	III	0	0	29	7	No
L6	IV	1	0	17	0	Yes
L7	III	5	2	4	1	Yes
L8	IV	0	0	29	1	No
L10	III	6	3	14	0	Yes
L11	IV	0	7	0	0	Yes
L12	IV	4	1	13	0	Yes
L13	II	0	0	22	0	Yes
L14	IV	0	0	16	0	No
L16	III	1	0	0	0	Yes
L17	IV	0	3	0	0	Yes
L18	IV	0	0	5	4	No
L19	IV	0	5	0	0	Yes

**Table 4:** The tabulation of all the detected mutations that are shared between tumor, CTCs and ctDNA in regardless of the differential time points for the breast cancer samples.

Patient	Disease stage at the point of blood collection	Shared mutations between Tumor and CTC	Shared mutations between Tumor and ctDNA	Shared mutations between CTC1 and CTC2	Shared mutations between CTC and ctDNA	Availability of tumor
B1	IV	9	7	18	1	Yes
B2	IV	10	9	12	2	Yes
B3	IV	5	8	13	6	Yes
B4	IV	1	1	17	0	Yes
B5	IV	15	9	28	0	Yes
B7	III	4	5	38	1	Yes
B8	II	8	5	37	1	Yes
B9	IV	5	6	10	1	Yes
B10	IV	0	4	5	1	Yes
B11	IV	10	9	26	2	Yes
B12	III	1	2	21	0	Yes
B13	IV	8	9	8	0	Yes
B14	III	10	12	14	5	Yes
B15	IV	3	2	0	1	Yes
B16	III	9	2	15	0	Yes
B17	IV	21	17	10	1	Yes
B18	III	3	23	0	2	Yes
B20	IV	11	6	23	2	Yes
B22	IV	3	0	30	0	Yes
B23	II	1	1	19	2	Yes
B24	IV	4	3	24	0	Yes

Of note, two lung cancer patients (L6 and L13) had more than 50% of the detected mutations found privately only in the CTCs and not in ctDNA. Both of these patients had a survival shorter than one year, indicating that the highly heterogeneous profiles of CTCs may provide information to stratify patients with poorer prognosis.

We also noted more private mutations in the CTCs and ctDNAs for breast cancer samples, at least partly due to increased number of CTCs and ctDNA analyzed in breast cancer as compared to lung cancer samples. We did not detect any clear trend for the degree of heterogeneity with patient survival outcome for breast cancer samples. Longer follow up and larger sample sizes will be required to investigate the association with survival outcome.

### CTCs and ctDNA Provide Complementary Genomic Information of Metastatic Tumour and Evolving Genetic Signatures During Disease Progression

**Figure 5:**
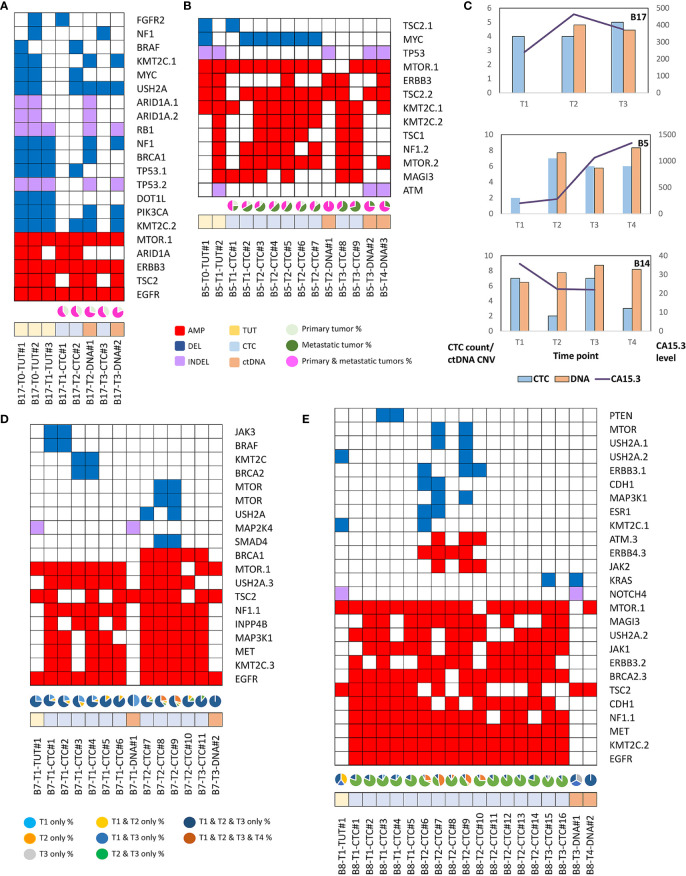
The mutation profiles and evolving genomics alteration in CTCs, ctDNA, primary and metastatic breast tumors. Red rectangles represent amplifications. Blue rectangles represent deletions. Green rectangles represent missense, Stopgain or Stoploss somatic SNVs. Purple rectangles represent somatic INDELs. TUT, CTC and DNA refers to tumors (yellow rectangles), individual CTC (blue rectangles) and ctDNA (orange rectangles) respectively. **(A)** Breast cancer patient, B17 with homogenous mutations profile in the primary (T0) and metastatic (T1) tumors. The pie chart represents the fraction of detected mutations to the primary tumor (light green) or metastatic (dark green) tumor or both tumors (pink). **(B)** Breast cancer patient, B5 with heterogenous mutations profile between primary and metastatic tumors. CTCs and ctDNA displayed better resemblance to metastatic than the primary tumors. **(C)** The CTC count, ctDNA CNV and CA15.3 level of serially collected samples for B5, B14 and B17 patients. **(D)** Majority of the mutations are consistently found at different time points in B7 breast cancer patient. However, selected mutations were only found in certain time points. The pie chart represents the fraction of detected mutations found at different time points. **(E)** Evolving genomics alterations during different course of treatment such as emergence of JAK2, ATM and KRAS mutations.

The mutation profiles of CTCs, ctDNA, matched primary and metastatic tumors of selected breast cancer patients were further compared spatially and temporally. Where the degree of mutational heterogeneity between paired primary and metastatic tumors is low, mutation profiles found in the CTCs and ctDNA were similar to the tumors (**Figure 5A**). In contrast, in primary and metastatic tumors that exhibited a high degree of mutational heterogeneity (**Figure 5B**), CTCs and ctDNA displayed higher degree of resemblance to metastatic tumor than the primary tumor, indicating that CTCs and ctDNA are potentially valuable resources to inform on the heterogeneity of metastatic tumor.

In order to investigate the temporal genetic signatures during the course of treatment and disease progression stages, we obtained serial collection of tumor, CTCs and ctDNA of different treatment cycles from a subset of breast cancer patients (n=15). The longitudinal follow up of CTC count and ctDNA CNV displayed differential correlation with the CA15.3 biomarker (**Figure 5C**). We found that the majority of DNA aberrations were consistently detected at different time points (**Figure 5D**). Notably, we observed several CNV such as *JAK3, BRAF or MTOR* amplifications were present only at selected time points of the collected CTCs but not found in the matched ctDNA. We posit that this observation could be explained by the viable CTCs that have evolved and survived the treatment while ctDNA provided genetic information of apoptotic tumor cells. Further, we found emerging of new *JAK2* and *ATM* alterations in patient B8 at the second time point (**Figure 5E**), indicating possible evolving tumor clonal growth during disease progression.

### The Mutation Profiles of CTC, ctDNA and Tumour Displayed Signatures Associated With Patient Survival Outcome and Disease Progression

**Figure 6:**
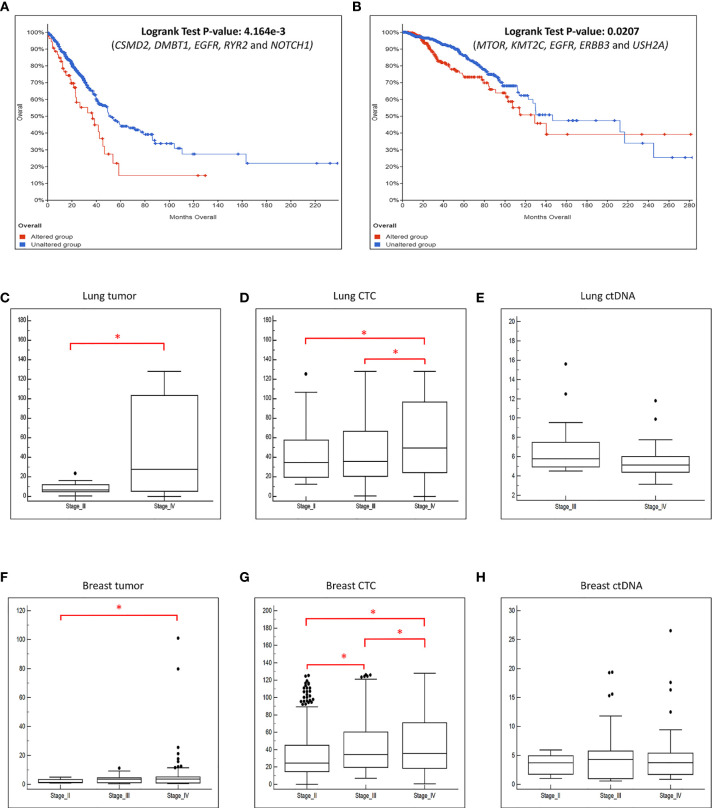
The frequently altered genes found in this study are associated with worse survival outcome. We performed survival analysis using data obtained from cBioPortal with a large cohort of **(A)** lung and **(B)** breast cancer patients. X-axis represents time to event. Y-axis represents overall survival probability. The patients with mutated genes (red) have significantly poorer survival compared to patients without mutations (blue). The boxplot of the CNVs detected in the **(C)** tumor, **(D)** CTC and **(E)** ctDNA grouped by the disease stage for lung cancer patients. The boxplot of the CNVs detected in the **(F)** tumor, **(G)** CTC and **(H)** ctDNA grouped by the disease stage for breast cancer patients. There is significant increase of copy number changes (*p-value ≤ 0.05, t-test) when the disease progressed in the tumor and CTCs.

We further explored whether the concomitant genomic alterations that present at high frequency in the tumor, CTCs and ctDNA, may provide information that is linked to patient survival outcome. Taking the top 5 most frequently concomitant altered genes detected in lung cancer CTC/ctDNA samples (*CSMD2, DMBT1, EGFR, RYR2* and *NOTCH1)* and breast cancer CTC/ctDNA samples *(MTOR, KMT2C, EGFR, ERBB3* and *USH2A)* from our study, we applied this gene set to a curated database of lung (n=511) or breast (n=996) cancers (13–21) obtained from cBioPortal (13, 14) for survival analysis. We found that these gene sets were significantly associated with worse survival outcome (**Figures 6A, B**). In addition, we found significant increase of copy number alterations in the CTC and tumor samples when the disease progressed (**Figures 6C–H**). The absence of such observation in ctDNA could be explained by its smaller sample size. Cumulatively, this indicates that the mutation profiling of CTCs and ctDNA is useful to provide genetic information with prognostic relevance.

## Discussion

CTCs and ctDNA represent different tumor components present in the circulation of cancer patients. Relatively few studies have analyzed both CTCs and ctDNA concurrently, hence their similarity or differences remains largely unknown. Though a few previous publications have analyzed CTCs, ctDNA and tumor from the same patient, these studies focused on new technology platforms and involved small number of patients (30, 31). For example, the work by Shaw et al. (30) isolated the CTCs using DEPArray based on epithelial marker from breast cancer patients, hence potentially missing the CTCs of mesenchymal phenotype that is critical during disease progression (20). Similarly, Sundaresan et al. utilized EPCAM-coated chip for CTC isolation, but only analyzed a single EGFR T790M mutation, and hence it lacked a more comprehensive evaluation of the genomic profiles of CTCs, ctDNA and tumor (31). In addition, these studies obtained CTCs and ctDNA from different tubes of blood, hence not accounting for possible inter-sample heterogeneity. Though the study by Manier et al. analyzed the genomic alterations of CTCs and ctDNA from patients with multiple myeloma (32) taken from the same source sample, the similar approach on other solid tumors is lacking. To address this, we obtained CTCs and ctDNA from the same source sample, and used label-free CTC isolation followed by CD45-negative selection of CTCs at single cell resolution with a new single cell isolation platform, DropCell, to provide agnostic selection regardless of epithelial or mesenchymal phenotype.

We had recently described the heterogeneity in gene expression of single CTCs relative to intra-tumoral heterogeneity, between metastatic and non-metastatic lung cancer (33), as well as comparing the drug-responsive and drug-resistant lung cancer lines (34). Here we found that the majority of the CTCs and ctDNA displayed similar mutation profiles with the matched tumor. However, we noted some private mutations that were found only in two or more CTCs from the same patients, consistent with other studies (30, 32). This highlights that CTC heterogeneity could be dependent on tumor heterogeneity and clonal selection. In contrast, we observed less heterogeneity in the ctDNA as compared to CTCs, indicating possibly that the ctDNA was derived largely from the dominant clone in the tumor. Intriguingly, patients with distant metastasis to multiple organs displayed a higher degree of heterogeneity in tumor and CTCs, suggesting that subclonal evolution in tumor progression also featured in CTCs. This was further supported by an evolving genomic signature in sequential samples of CTCs and ctDNA. Hence, the ability to characterize the cells with metastatic properties could provide useful insights for better management of cancer in patients. Future studies would be required to define the metastatic heterogeneity of the CTCs and the association with poorer survival in tumors exhibiting the heterogeneity observed here.

Finally, frequent genetic alterations detected in lung and breast cancer have been reported by previous studies to be associated with poor prognosis in lung or breast cancer patients (35–43). Accordingly, we found that the top five most frequently mutated genes in both CTCs and ctDNA had prognostic value when applied to existing cancer cohorts. Indeed, the alterations found in these genes such as *RYR2, NOTCH1 and DMBT1* have strong association with high tumor burden and tumorigenesis (44–47), possibly resulting in a worse prognosis in lung cancer patients. Further, frequent alterations of *USH2A, EGFR* and *ERBB3* were previously reported in association with cellular proliferation (48–51). Further, we observed significant increase of copy number changes in the tumor and CTCs during disease progression. This finding suggests that incorporating the mutational profile for the CTCs in addition to enumeration, could build upon existing prognostic variables defined by FDA approved systems such as CellSearch, and potentially better defining the groups that need to receive adjuvant therapy.

Besides molecular characterization, the functional analysis of the identified mutations could provide additional insights to assess the consequences of these mutations in cancer therapy. We have previously developed CORTAD-seq that allows for concurrent analysis of transcriptome and mutation at single cell resolution (34). Using this tool, we found that single cell that harbors selected tyrosine kinase inhibitor (TKI)-responsive or -resistance EGFR mutations possesses differential transcriptomic signatures upon TKI stimulation. Hence, we envision that future study incorporating CORTAD-seq or other similar tool will be useful to dissect the consequences of mutation on cellular phenotypes.

We acknowledge that a technical limitation in our study is the low number of CTCs that passed the quality control check. Only ~34% of all the isolated lung CTCs and ~68% of all the isolated breast CTCs were kept in the final analysis (**Supplementary Table 6**). This observation is in concordant with the findings by Dirix’s and Park’s laboratories where 43 to 60% of the isolated CTCs yielded positive result from the downstream transcriptional and mutational analysis (52, 53), highlighting that it is important to improve the quality of the amplified DNA from the CTCs sample. We believe that this is a technological limitation that can be overcome with improving single cell analytical platforms that could be mated with this workflow.

Cumulatively, we present a workflow for robust simultaneous evaluation of CTCs and ctDNA from the same source sample. The molecular profiling of serially collected liquid biopsies could inform and build on existing algorithms for prognostication and management using CTCs and ctDNA.

## Data Availability Statement

The original contributions presented in the study are included in the article/**Supplementary Material**. Further inquiries can be directed to the corresponding authors.

## Ethics Statement

All patients gave written informed consent to participate in this study and the biological samples were collected from the patients following the Institutional Review Board (IRB) approval. The patients/participants provided their written informed consent to participate in this study.

## Author Contributions

SK, JT, LP, HP, and YH performed the experiments described in this study. XL, SK, LP, and WL performed the data analysis. ST, TY, and YC processed the CTC enrichment and single cell isolation experiment with the technical support from YL. RN, KL, MA, QN, DT, W-TL, and YY recruited patients for this study. CL, TL, and AH provided guidance and support to this work. SK wrote the manuscript with the inputs from XL, ST, W-TL, and YY. All authors contributed to the article and approved the submitted version.

## Funding

This work is supported by the Career Development Award (14302FG096) by Joint Council Office (JCO), Agency for Science, Technology and Research, Singapore to SK; Young Individual Research Grant (NMRC/OFYIRG/0056/2017) by National Medical Research Council (NMRC), Singapore for SK; Clinician Scientist Award (MOHIAFCat1/0034/2015 and CSA-INV/0025/2017) by NMRC, Singapore for W-TL; Clinician Scientist Award (MOH-CSAINV18nov-0009) for YY by NMRC; the infrastructure of Lung Cancer Consortium Singapore that is jointly funded by Open Fund Large Collaboration Grant (NMRC/OFLCG/002/2018) by NMRC, Singapore; Trailblazer Foundation, Singapore Millennium Foundation, and the National Cancer Centre Research Fund.

## Conflict of Interest

ST is the current employee of Sysmex and ex-employee of Clearbridge mFluidics Pte Ltd. YL is ex-employee of Biolidics Ltd. CL is a cofounder and shareholder of Biolidics Ltd.

The remaining authors declare that the research was conducted in the absence of any commercial or financial relationships that could be construed as a potential conflict of interest.
